# Phase-contrast zoom tomography reveals precise locations of macrophages in mouse lungs

**DOI:** 10.1038/srep09973

**Published:** 2015-05-12

**Authors:** Martin Krenkel, Andrea Markus, Matthias Bartels, Christian Dullin, Frauke Alves, Tim Salditt

**Affiliations:** 1 Institute for X-ray Physics, University of Göttingen, 37077 Göttingen, Germany; 2 Department of Haematology and Medical Oncology, University Medical Center Göttingen, 37075 Göttingen, Germany; 3 Department of Diagnostic and Interventional Radiology, University Medical Center Göttingen, 37075 Göttingen, Germany; 4 Department of Molecular Biology of Neuronal Signals, Max-Planck-Institute of Experimental Medicine, 37077 Göttingen, Germany

## Abstract

We have performed x-ray phase-contrast tomography on mouse lung tissue. Using a divergent x-ray beam generated by nanoscale focusing, we used zoom tomography to produce three-dimensional reconstructions with selectable magnification, resolution, and field of view. Thus, macroscopic tissue samples extending over several mm can be studied in sub-cellular-level structural detail. The zoom capability and, in particular, the high dose efficiency are enabled by the near-perfect exit wavefront of an optimized x-ray waveguide channel. In combination with suitable phase-retrieval algorithms, challenging radiation-sensitive and low-contrast samples can be reconstructed with minimal artefacts. The dose efficiency of the method is demonstrated by the reconstruction of living macrophages both with and without phagocytized contrast agents. We also used zoom tomography to visualize barium-labelled macrophages in the context of morphological structures in asthmatic and healthy mouse lung tissue one day after intratracheal application. The three-dimensional reconstructions showed that the macrophages predominantly localized to the alveoli, but they were also found in bronchial walls, indicating that these cells might be able to migrate from the lumen of the bronchi through the epithelium.

Lung tissue, with its intricate three-dimensional (3D) system comprising the bronchial tree, alveoli, and blood vessels, is an excellent example of how the 3D structures of tissues enable their physiological function. Conversely, structural alterations are associated with different pathological states. To investigate these relationships quantitatively, the 3D structure of the tissue must be assessed from the cellular to the organ scale. Furthermore, cell-tracking studies are of high interest for the location of cells in relation to anatomical structures. The conventional approach of sectioning histology followed by optical microscopy or electron microscopy is associated with several major deficits and restrictions. Apart from possible slicing or staining artefacts, it is extremely tedious and time-consuming to record an entire organ or large field of view (FOV), making it almost impossible to cover the complete 3D tissue architecture of many different specimens, even at moderate resolution.

In the use of 3D biomedical imaging to fill this gap, x-rays are the first choice due to the required penetration depth and resolution power. X-ray tomography is a powerful technique for imaging high-density (‘hard’) structures in tissues and bodies that can otherwise only be visualized in a destructive manner. However, the advantage of transparency for hard x-rays is also a considerable drawback for the examination of most non-absorbing (‘soft’) tissues, resulting in a lack of contrast for low-density tissue. For micron- and nanometre-scale structures, low absorption coefficients become even more restrictive because measurable absorption levels build up only over longer path lengths. A closer examination, however, of the optical constants involved in the x-ray index of refraction 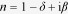 shows that, for the elements and photon energies relevant for the tomography of tissues, the refractive decrement 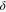 is up to three orders of magnitude larger than the absorption component 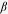. Even if only relative changes, e.g., between water and protein, are relevant for the imaging, this opens up a huge potential to increase the contrast and resolution for soft tissues^1^,^2^,^3^. This alteration can be achieved if interaction via 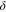 is exploited, i.e., if the phase shift 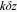 of an x-ray wave with wave number 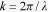 propagating through matter over a distance 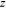 can be visualized. See^4^,^5^ for a review.

To this end, several phase-contrast techniques have been developed in recent decades^6^,^7^. The most important phase-contrast principles are Zernike phase-contrast in zone plate x-ray microscopes^8^,^9^, grating interferometry^10^,^11^,^12^, scanning diffraction microscopy^13^,^14^, and phase-contrast formation based on the free propagation of the x-ray beam behind the sample^15^,^16^,^17^. Each technique has its advantages and drawbacks, and each can be applied to a certain range of length scales and in certain types of applications^18^,^19^. For zone plate-based Zernike phase-contrast, resolutions down to ten nanometres have been achieved in 2D^18^, but a resolution in this range cannot be achieved in tomography of thick specimens^9^. Furthermore, the low efficiencies of the optical elements behind the sample (zone plate, phase annulus) lead to the need for an increased radiation dose, and the calculation of quantitative phase-contrast values is typically based on idealized assumptions for the optical elements. Grating or analyser-based phase-contrast are the methods of choice for macroscopic field of view, with maximum-resolution values of approximately 4 μm^20^. Coherent lensless imaging methods are more dose efficient because no optical element is needed behind the sample for image formation. Scanning transmission x-ray microscopy (STXM) with ptychographic phase reconstruction^21^ and coherent diffractive imaging^22^,^23^ have both reached a resolution of approximately 10 nm in 2D for strongly diffracting test structures and sub-50 nm resolution for single cells. However, a major drawback of these methods is the limited FOV resulting from the small beam size and the scanning overhead. On the organelle^23^ and single-cell levels^24^,^25^, high-resolution 3D reconstructions have been achieved, and at even finer scales, the FOV can be enlarged in tomographic ptychography^26^. However, this increased resolution comes at the cost of long measurement times in the order of 10 h, which limits the usefulness of this method for objects in the range of several hundred μm.

Propagation-based phase-contrast enables a lensless full-field imaging approach compatible with a wide range of sample sizes, resolution values, photon energies and source characteristics. The imaging scheme is conceptually simple: extending the standard radiographic exposure by a free propagation distance between object and detector and enhanced (spatial) coherence enables a defocused image based on self-interference (in-line hologram) to be recorded, which still bears recognizable similarities with the object. The main difficulty is the phase-retrieval step, which is typically based on *a priori* information regarding the sample (e.g., a compact support^27^) or on idealized assumptions (e.g., weakly or non-absorbing objects^28^) often combined with intensity measurements at different Fresnel numbers (e.g., different defocus distances)^29^. In x-ray waveguide-based (cone beam) holographic imaging, we have previously demonstrated resolutions below 30 nm^30^,^31^ and fields of view in the range of 100 μm as well as tomographic 3D reconstructions with nanometre resolution of single cells^32^. Propagation-based phase-contrast tomography of larger tissue samples has also been reported^33^,^34^,^35^,^36^.

For the purposes of imaging tissues from several mm up to entire small animal organs, at a resolution in the range of 100 nm to several microns, x-ray propagation imaging is the method of choice. However, 3D visualization of a whole organ such as the lung with nanoscale resolution would require handling unreasonable amounts of data. A suitable approach must therefore allow for 3D imaging of a large FOV on the millimetre scale with the possibility of zooming in to regions of interest (ROIs), yielding information at the nanometre scale to visualize sub-cellular features. Propagation-based phase-contrast in cone beam geometry enables tomography with an effective zoom function as controlled by the focus to object distance 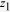 and the object to detector distance 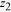
32,34,37,38, but this method has thus far not been applied to examine biological hydrated soft tissue.

In this work, we have used propagation-based phase-contrast with zoom tomography to measure the 3D density distribution of hydrated lung tissue samples in a large FOV and at high magnification with sub-cellular resolution. To achieve the enhanced nanoscale resolution and high dose efficiency, we have tuned the characteristics of the incoming wave-front (coherence, curvature, reduced aberrations) by mode filtering based on x-ray waveguides, which act as a secondary quasi point source for object illumination. Using this approach, we have then visualized the distribution of barium-labelled macrophages in healthy and asthmatic lung tissues. Although macrophages are known to be involved in allergic inflammation^39^, the exact role of macrophages in asthma is still not well understood^40^,^41^,^42^,^43^. To investigate this role, the distribution of macrophages and their migration properties within the lung are important factors requiring 3D visualization with high resolution and contrast. Using these unprecedented phase-contrast tomography capabilities, we can detect barium-labelled murine alveolar macrophages of the cell line MH-S^44^ in asthmatic and healthy lung tissue one day after intratracheal (i.t.) application. We show that macrophages localize predominately to alveoli and are able to penetrate the epithelial layer between the airway lumen and parenchyma. In addition, we demonstrate the dose efficiency and scalable resolution capability of this approach by providing 2D reconstructions of single living macrophages, with and without phagocytized contrast agents.

## Experimental Procedure

**Figure 1:**
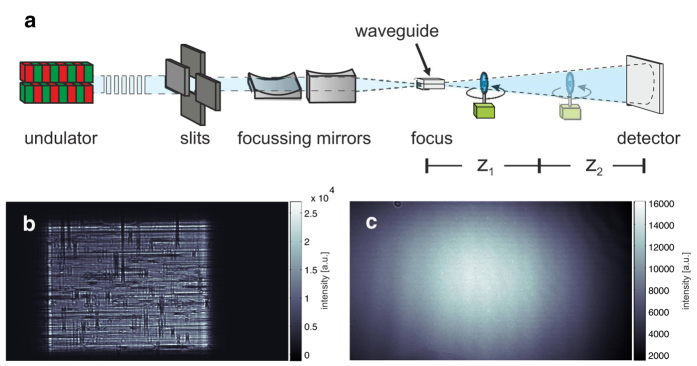
(**a**) Schematic of the imaging geometry: The x-ray beam is generated in an undulator at a 3rd-generation synchrotron storage ring. A secondary source is created by focusing the beam with KB mirrors, yielding a divergent beam behind the focus, which makes it easy to control the magnification by changing the defocus distance. The radiation is detected by a scintillation-based detector. (**b**) A typical KB farfield showing wavefront artefacts due to mirror figure errors. The use of an x-ray waveguide for mode and coherence filtering and for further reduction of the source size down to 20 nm results in a significantly improved wavefront for the imaging experiment (see (**c**) for a typical farfield of the waveguide beam).

Barium sulphate-labelled murine alveolar macrophages of the cell line MH-S^44^ were instilled i.t. in asthmatic and control mice 24 h prior to euthanasia. Cell labelling was achieved by adding a suspension of barium sulphate (BaSO_4_) directly to the media followed by co-incubation. Lung tissue slices were prepared and mounted in phosphate-buffered saline (PBS) between two thin foils immediately before the tomographic measurements. Two different synchrotron setups with divergent beams obtained with Kirkpatrick-Baez (KB) or waveguide (WG) illumination were used for the experiments, resulting in different illuminating beams. The setup is sketched in Fig. 1(a) with resulting farfield intensity distributions shown in Fig. 1(b) for the KB and in (c) for the WG setup, both obtained for comparison at the P10 beamline.

## Results

### Holographic imaging of asthmatic mouse lungs

**Figure 2:**
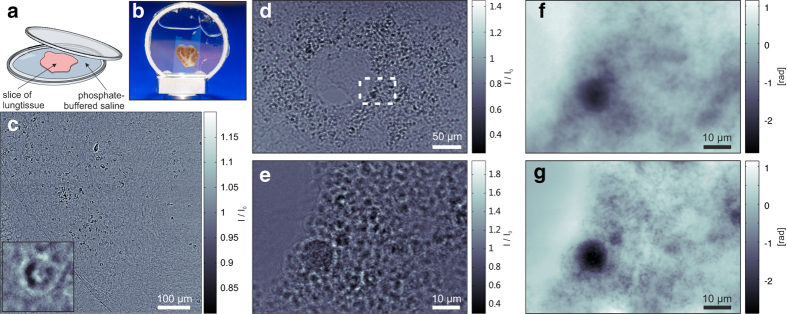
Sample preparation and measurements: (**a**) Sketch of sample mounting. The 500 μm thick mouse lung tissue slices were placed in buffer solution on a thin foil and covered with a second foil. (**b**) Photograph of a final prepared sample mounted on a sample holder. (**c,d,e**) Phase-contrast projections of lung tissue samples corresponding to single projections out of the tomographic scans shown in Fig. 3 to 5: (**c**) A typical empty beam-corrected phase-contrast image recorded at the ID22 beamline showing direct contrast of the lung structure. The inset shows a detailed view scaled to have the same pixel size as (**e**). (**d**) A typical empty beam-corrected phase-contrast image recorded at the P10 beamline. Due to the smaller Fresnel numbers, the image is of holographic nature, showing less correspondence to the real spatial structure. (**e**) Phase-contrast image at higher geometric magnification (smaller defocus distance 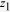) of the area marked by a dashed rectangle in (**d**). (**f**) Reconstructed phase distribution out of (**d**) showing the region inside the dashed rectangle. (**g**) Reconstructed phase out of (**e**) and three other distances showing the higher resolution of the zoomed dataset.

Figure 2 shows three typical empty beam-corrected images of lung tissue samples with labelled macrophages obtained at the ID22 (Fig. 2(c)) and at the P10 beamline, showing a projection for a large FOV (Fig. 2(d)) and the same projection angle at a larger magnification (Fig. 2(e)). Depending on the Fresnel number 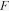, different imaging regimes are covered, ranging from the direct contrast regime (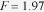) in Fig. 2(c) to a strong holographic regime (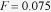) in Fig. 2(e). The measured intensity in the direct contrast regime is explained by the 2D derivative (Laplacian) of the phase distribution behind the sample. Although nearfield phase-retrieval algorithms based on, e.g., the transport of intensity equation (TIE)^45^ would still be feasible in this imaging regime, we used a more general method based on the contrast transfer function (CTF), which has been described previously^46^. The TIE approximation of small propagation distances fails for larger image frequencies, and the CTF approach delivers results with higher resolution. As an example, 2D phase reconstructions are shown in Fig. 2(f) and (g) and Supplementary Figs. 1 to 3 with corresponding parameters shown in Supplementary Table 1. Phase retrieval was performed individually for each projection of a tomographic scan, and subsequent tomographic reconstruction yielded a 3D dataset.

**Figure 3:**
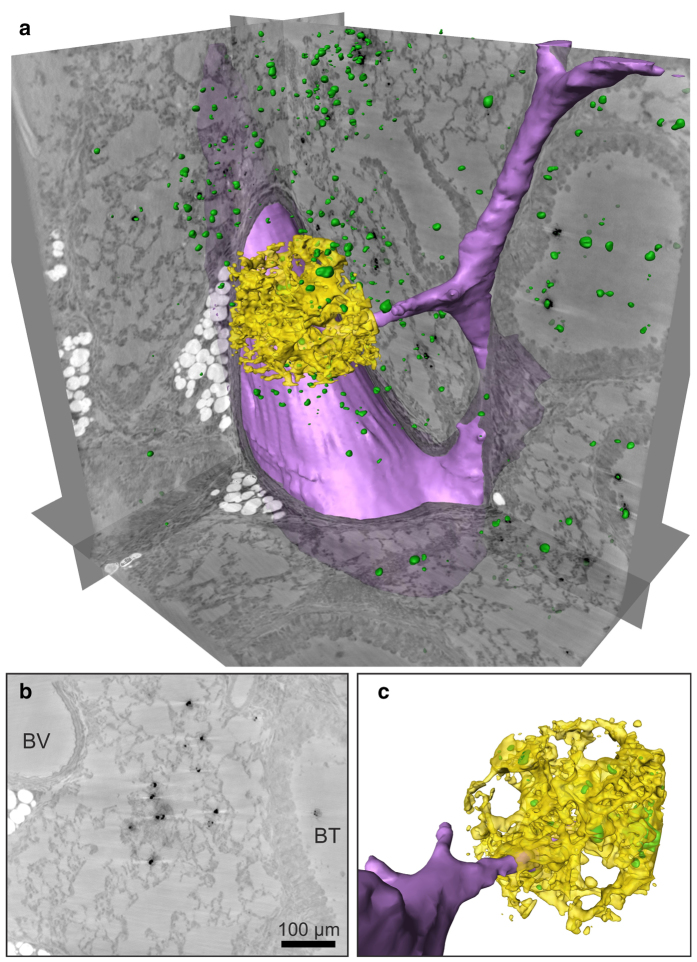
Results obtained in the KB setup: (**a**) 3D rendering of a reconstructed asthmatic lung tissue volume showing three orthoslices together with automatically labelled (density based) barium clusters (green) and alveolar walls in a small ROI (yellow). A part of a blood vessel was marked semi-automatically (purple). (**b**) Orthoslice perpendicular to the tomographic rotation axis through the reconstructed volume, as obtained from a single distance tomogram with voxel size 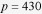 nm. Darker values correspond to denser material. Barium sulphate particles (black) and fat (white) show a strong density contrast compared to soft tissue. A blood vessel (BV) and a bronchial tube (BT) can be identified based on their different wall morphologies. (**c**) Close-up of the ROI from a different viewing angle.

Figure 3 depicts the results of a reconstructed 3D volume of a lung tissue slice, recorded with KB illumination in the direct contrast regime at ID22. The rendering in Fig. 3(a) shows three orthoslices: a semi-automatically segmented blood vessel (purple), automatically labelled soft tissue (half transparent yellow) in a ROI in the centre of the volume, and automatically labelled barium sulphate (green). Several bronchial tubes can be seen with a typical wall morphology showing single goblet cells protruding into the airways. A single planar orthoslice is shown in Fig. 3(b). The complete information contained in the datasets can be assessed from a video of the 3D rendering provided as Supplementary Video 1. With the high flux available at the ID22 beamline, a tomographic scan takes only approximately 15 minutes. A total of nine successful tomographic scans were recorded from five different mice: two asthmatic mice, two control mice and one blank specimen without instilled macrophages (see Supplementary Fig. 5). By visual inspection, the bronchial walls were found to be slightly thicker in asthmatic compared to control mice, but the location and distribution of macrophages did not seem to differ. In the reconstructed volume, the macrophages can be readily rendered based on automatic segmentation of the barium inside the cells. The 2D orthoslices show that barium- labelled macrophages are found predominantly within alveoli (see Fig. 3(b)). However, only a full 3D analysis can unambiguously distinguish between an adhering and an embedded location. Thus, we have rendered the epithelium together with the macrophages, based on automatic segmentation in a small ROI in the centre of the reconstruction volume, as shown in Fig. 3(a), as well as from a different viewing angle in (c). The positions of the labelled macrophages suggest that many macrophages are surrounded by soft tissue. To investigate this location in further detail, it is necessary to increase the resolution. Note that the present dataset was recorded at a voxel size of 430 nm and is rendered at 860 nm (2 × binning) in Fig. 3. To obtain high-resolution reconstructions, we used a setup based on waveguide optics, as described below.

### Zoom tomography using the waveguide setup

**Figure 4:**
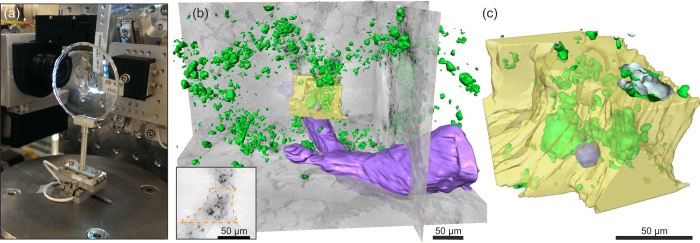
Large FOV results obtained with the waveguide setup: (**a**) photograph of the sample mounted to the sample holder. (**b**) 3D rendering of the reconstructed volume of a control lung showing 3 orthoslices together with automatically labelled BaSO_4_ particles (green), a semi-automatically rendered blood vessel (purple), a manually labelled bronchial wall inside a ROI (yellow), and the contours of a single macrophage in this ROI (blue). An orthoslice through the area used to segment the bronchial wall (dashed orange line) is shown in the inset. (**c**) Close-up the segmented ROI viewed from a slightly different angle. The same area is measured with a larger magnification, shown in Fig. 5.

**Figure 5:**
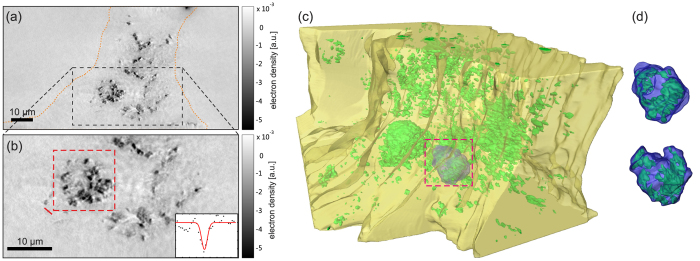
Zoomed-tomography results obtained with the waveguide setup: The sample shown in Fig. 4 was moved closer to the effective source, resulting in a higher geometric magnification. (**a**) Slice through the 3D volume showing the area used for segmentation of the bronchial wall (dashed orange line). (**b**) Close-up of the area of the dashed rectangle shown in (**a**). A profile through the solid line is plotted in the inset showing a feature size of 249 nm FWHM. The area marked by the red dashed rectangle shows the cell, which is rendered in blue. (**c**) 3D rendering of the data, showing automatically labelled aggregates of 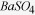 particles (green), the manually labelled bronchial wall (yellow), and a manually labelled cell outline (blue). (**d**) Close-up of the barium-labelled cell marked by pink dashed lines in (**b**) and (**c**) from two different viewing angles showing the internal barium distribution.

Figure 4 shows the results obtained with the waveguide setup at the P10 beamline with the same colour coding as in Fig. 3. Barium within the instilled macrophages was automatically labelled and is rendered in green. A semi-automatically segmented blood vessel is shown in purple. A bronchial tube (not rendered) that branches into two tubes lies parallel to the blood vessel. To provide a detailed impression of the 3D volume, a video of the rendering is available as Supplementary Video 2. A small part of the bronchial wall, marked by the dashed line in the inset of Fig. 4(b), was manually segmented and is rendered in half-transparent yellow. The outline of a single barium- labelled macrophage inside this ROI was manually segmented, and it is shown in blue (Fig. 4(c)). By moving the sample closer to the effective source, from 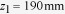 to 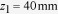, we increased the magnification while keeping the central ROI in the reconstruction volume. A second tomographic scan at this zoom setting was performed using the CTF-based reconstruction algorithm based on a four-distance dataset. For each projection, phase retrieval was performed using the resampled and aligned images, followed by tomographic reconstruction, as described in detail in Supplementary Methods 1 and 2. A rendering of the resulting 3D volume is shown in Fig. 5(c), in which the same bronchial wall segmentation and cell outline as in Fig. 4(c) are shown together with automatically labelled barium in green. A detailed video of the rendering is available as Supplementary Video 3. The macrophages are clearly located inside the bronchial wall, indicating that macrophages are able to migrate through the bronchial epithelium.

### Dose and resolution

The higher resolution of the zoomed tomograph in Fig. 5 immediately becomes apparent upon inspection of the barium inside the macrophages. Rather than a homogeneous density, we find individual barium clusters scattered within the cell. The macrophages can be identified by segmenting the envelope around the barium (see the cell rendered in blue), which is exemplified in the dashed red rectangle in (c), as well as in isolated form in (d). The orthoslices in Fig. 5(a,b) correspond to a cut through the same macrophage. A Gaussian fit through a single small particle in this orthoslice reveals a feature size of 249 nm (see the inset in Fig. 5(b)). Fourier shell correlation indicates a resolution in the 3D dataset of approximately 170 nm using a half-bit criterion according to^47^ (see Supplementary Methods 3 for details). Thus, the resolution was increased compared to the results obtained in the KB setup at ID22, but it was significantly worse than the sub-30 nm resolution achieved using the same waveguide setup with two-dimensional test patterns^30^. This resolution loss can be attributed to small drifts that occur during the time of the tomographic scan, as the dose values (see Table 1) are comparable to those in^30^. We expect that the resolution might be increased by improving the mechanical stability or by using optimized alignment algorithms.

Fluence and dose values for the datasets shown are listed in Table 1. Zoomed tomography at the ID22 beamline was impeded by radiation damage resulting from the higher dose at short defocus distance. Comparing the WG (Fig. 4) with the KB setup (Fig. 3), the dose applied to the sample was 18 times lower, although it was measured at a slightly higher resolution. Note that in ptychographic coherent diffraction imaging, a dose of 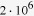 Gy was required for 3D reconstruction at a similar resolution level^14^. Thus, the dose was 20 times higher than in the zoomed WG dataset shown here.

### Imaging of living single cells

Motivated by the dose-efficient imaging results for lung tissue, we sought to demonstrate the ability of the method to visualize living macrophages. Apart from being a proof of concept, it was of interest to study in more detail how the barium-based contrast agent is distributed within the cell.

**Figure 6:**
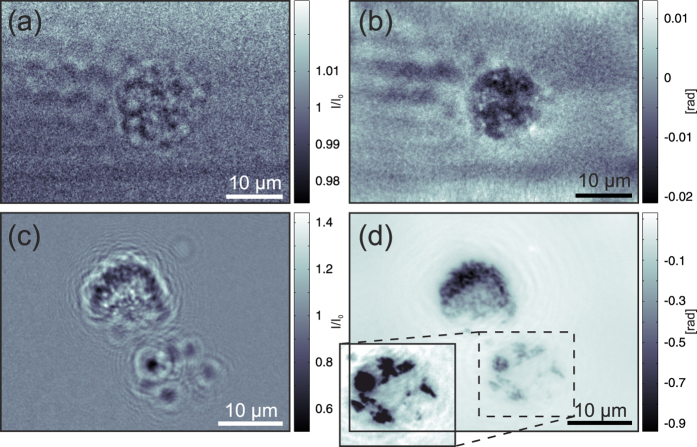
Living single-cell imaging at P10: Living macrophages were kept in medium in an ibidi flow chamber that was mounted onto a sample holder. The left column shows empty beam-corrected phase-contrast images of (**a**) an unlabelled macrophage and (**c**) two barium-labelled cells. The right column shows (**b**) the corresponding phase reconstruction of (**a**) using 3 distances, each 10 × 1 s, and (**d**) the phase reconstruction of (**c**) using 5 distances, each 1 s. The inset shows the lower right cell with contrast adjusted to enhance the weak signal of the cell structure.

To this end, single macrophages were kept in microfluidic chambers (ibidi microscopy slide) and transported in a mobile incubator to the beamline. The chambers were mounted at 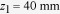 behind the effective source, with macrophages adhering to the windows, which are transparent for both 13.8 keV x-rays and visible light, facilitating alignment in the beam. Figure 6 shows holographic phase-contrast images and their corresponding phase reconstructions for a native unlabelled cell (a,b) and for cells that were labelled with the contrast agent by co-incubation with a barium sulphate suspension (c,d). This result proves that single cells can be resolved without contrast agents and shows that the strong contrast in the 3D reconstructions shown in Fig. 3 to Fig. 5 results mainly from the contrast agent. The inset in Fig. 6(d) shows that at low barium concentrations, the cell structure can be observed, although the much stronger contrast of the barium affects the image impression. We also observed that barium sulphate particles are not homogeneously distributed in the cell but, rather form aggregates mainly at the periphery. This finding is in agreement with the 3D reconstructions. Depending on the resolution of the image, this clustering of barium sulphate particles can complicate the interpretation of the 3D data.

## Summary and Discussion

In summary, we have demonstrated that holographic phase-contrast tomography can (i) reveal the 3D structure of hydrated mouse lung tissue slices down to the sub-cellular level and (ii) visualize barium-labelled macrophages within anatomical structures. Using a geometric zoom, imaging at a large field of view in the range of several 100 μm and at high resolution down to 170 nm for selected ROIs was achieved for the same tissue sample without deterioration of its structure. To exploit this zoom capability for soft biological tissues, high dose efficiency is required. We have achieved this efficiency by using x-ray waveguide optics that better fulfil the idealized assumptions on the illuminating wave-field regarding coherence, point-source geometry, and the nearly aberration-free wavefront.

Compared to ptychography, we have obtained reconstructions with a 20 times lower dose at similar resolution. We expect that with sophisticated alignment algorithms and/or faster accumulations, the resolution could be increased even further without increasing the dose. At the same time, the large overhead in data accumulation by scanning microscopy techniques (such as ptychography) are avoided in the present full-field imaging scheme. Naturally, this approach can still be combined with moderate scanning to further enlarge the FOV, which should make it possible to visualize the entire lung of a mouse in 3D. Compared to grating-based phase-contrast imaging, which can also easily cover large FOVs, propagation imaging offers significantly higher resolution down to the sub-cellular level and does not require any optical elements between the sample and the detector.

Persistent challenges in the phase-retrieval step have kept the advantages of propagation imaging from being fully exploited. In particular, in the holographic regime, conventional phase retrieval based on the TIE equation is no longer valid. As detailed in Supplementary Methods 1, we found that the approach based on the contrast transfer function (CTF) pioneered by Cloetens^15^ provides a rapid and robust means to achieve high-quality reconstructions (regarding contrast and resolution) even for low-contrast tissues if the following conditions are met: (i) data are recorded in the holographic regime to optimize contrast transfer for a broad range of spatial frequencies, (ii) the wavefront is sufficiently coherent and well controlled, and (iii) the image series with frames recorded at different magnifications and in different fields of view is well aligned. Further improvements, and in particular contrast values yielding quantitative electron density, can be achieved by extending the phase retrieval to iterative reconstruction algorithms.

Armed with the above imaging methodology, important biomedical questions can now be addressed, which are difficult to address with conventional histology. In particular, phase-contrast zoom tomography could become a novel tool for tracking cells in relation to anatomical structures. The present study on lung tissues in asthmatic and control mice has addressed the distribution of instilled barium-labelled macrophages within the lung. The 3D reconstructions obtained in this study show that one day after i.t. application of MH-S cells, these macrophages were located within the alveolar lumen and within bronchial walls. The low dose required also enabled the imaging of living single cells. Aggregated particles of precipitated barium sulphate were observed in the cytosol of the macrophages. Future experiments will include more sophisticated contrast agents that bind specifically to functional sites inside the macrophages at the organelle level.

## Methods

### Sample preparation

The immortalized mouse alveolar macrophage cell line MH-S (American Type Culture Collection, ATCC, USA) was maintained in RPMI medium supplemented with 10% FCS, 0.05 mM 2-mercaptoethanol and 2.06 mM glutamine^44^ in a humidified atmosphere at 5% CO_2_ and 37 °C. The cells (1 × 10^6^ cells/ml) were loaded with barium sulphate particles by co-incubating the cells for 4 h in standard media containing 3.5 μl/ml of the clinical contrast agent Micropaque CT (7.5 g BaSO_4_/150 ml) (Guerbet, France). The uptake of BaSO_4_ differed randomly among cells, but the amount used ensured that most cells would take up enough particles for robust cell tracking. A conventional OVA-induced asthma model was used as previously described^48^. All experiments were carried out in strict accordance with the guidelines for the care and use of laboratory animals of the local ethics office of the University Medical Center Göttingen. This study was approved by the Committee on the Ethics of Animal Experiments of the Niedersächsisches Landesamt für Verbraucherschutz und Lebensmittelsicherheit (LAVES) (Permit Number: 33.12-42502-04-12/0834). All painful procedures were performed under anaesthesia, and all efforts were made to minimize suffering. A total of 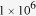 barium-labelled macrophages in 30 µ l PBS were i.t. instilled into the lungs of asthmatic and control BALB/c mice 72 h after the last OVA challenge under Ketamine 10% / Xylazine 2% anaesthesia. Mice were euthanised 24 h after macrophage instillation using an isoflurane overdose. Lungs were dissected and fixed in 4% paraformaldehyde for 24 h, and individual lung lobes were embedded in 5% agarose. Slices 500 µm in width were cut using a Leica VT1000 S vibrating blade microtome (Leica, Wetzlar, Germany) and they were stored in 0.02% sodium azide/PBS solution at 4 °C. Shortly before the measurements at the beamline, the slices were placed between two thin polypropylene foils mounted on 500 μm thick aluminium rings; see Fig. 2. To prevent drying during measurement, the sample holder was sealed. The two rings formed a closed chamber 500 μm thick. The samples were mounted vertically on sample holders, which fit the corresponding instrument. Photographs of the final mounted samples can be seen in Fig. 2(b) and Fig. 4(a). For imaging of living cells, microscopy slides (ibidi) were used as closed chambers to transport barium-labelled and unlabelled MH-S cells in CO_2_ independent medium to the beamline, keeping the cells at 37 °C for approximately 4 to 6 h in a mobile incubator.

### KB-based imaging at ID22

The first part of the experiments was carried out at the ID22NI beamline at the European Synchrotron Radiation Facility (ESRF) in Grenoble, France^49^. The x-rays were generated in an undulator and not further monochromatized (pink beam), leading to a very high flux in the order of 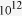 photons per second. The photon energy was set to 17.5 keV, with a relative bandwidth 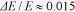. The beam was focused by two elliptically bent Kirkpatrick-Baez (KB) mirrors to a spot size of less than 100 nm in both directions, resulting in a divergent beam (8.1 vertical and 8.2 horizontal mrad divergence) that allows tunable magnification by changing the defocus distance 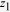 (see Table 1). The effective source size theoretically determines the maximal resolution possible with this setup. No additional optical element was placed in or behind the focus. A fully motorized sample tower positioned the air-bearing rotation stage. A scintillation-based detector was placed at 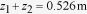 behind the focal plane. The detector was a high-resolution x-ray microscope based on a thin single-crystal scintillator viewed with a 10-fold objective lens. The images were captured with a fast read-out low-noise CCD camera (Frelon, ESRF)^50^ resulting in a detector pixel size of *p* = 0.756 μm. The propagation can be fully described by a single parameter, the Fresnel-number 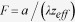, with the wavelength 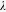, the effective propagation distance 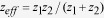 and a typical feature size 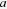. In the present work, we defined a typical feature size of ten pixels 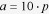.

### Waveguide-based imaging at the P10

To further increase the resolution, the second part of the experiment was carried out at the GINIX instrument installed at the P10 beamline at DESY in Hamburg, Germany. The x-rays were generated in an undulator, and the energy was set to 13.8 keV. The x-rays were monochromatized by a Si(111) double-slit monochromator, resulting in a relative bandwidth 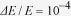. The x-rays were focused by KB mirrors to approximately 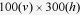 nm^2^. For high-resolution imaging, the effective source size was further reduced by precisely aligning an x-ray waveguide (WG) system consisting of crossed planar waveguides with an optimized two-component (Mo/Ge) cladding^51^ in the KB focus. By multi-modal interference, the waveguide beam propagated through the 59 nm thick carbon guiding layer over a total length of 0.76 mm, resulting in a reduced beam size in the exit plane of 16 nm FWHM, as determined by iterative inversion of the measured farfield data^31^,^52^, confirming the finite difference simulations carried out for optical design. The sample was positioned in the defocus of the beam at a distance 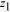 behind the waveguide exit on a fully motorized sample tower, including an air-bearing rotation. A high-resolution sCMOS x-ray camera (Photonic Science) was used in which a 15 μm thick Gadox scintillation screen was directly coupled via a 1:1 fibre-optic plate to the sCMOS chip, resulting in a detector pixel size of 6.54 μm. The detector was placed at 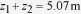 behind the effective source created by the WG. Figure 1 shows a typical farfield image of the pure KB beam without the waveguide (b) compared to a farfield image obtained after aligning the waveguide in the focus (c). As the figure shows, the divergence increased, reflecting the smaller source size, and the illumination exhibited fewer high-frequency artefacts. Due to the waveguide geometry and absorption in the cladding of the waveguide, the total flux was reduced by approximately two orders of magnitude.

## Author Contributions

F. A., T. S., C. D. and M. K. designed the experiments. A. M. prepared the samples. M. K., M. B. and T. S. performed the experiments. M. K. performed the analysis and prepared the figures. M. K. and T. S. wrote the main manuscript text. All authors reviewed the manuscript.

## Additional Information

**How to cite this article**: Krenkel, M. *et al*. Phase-contrast zoom tomography reveals precise locations of macrophages in mouse lungs. *Sci. Rep.* **5**, 9973; doi: 10.1038/srep09973 (2015).

## Supplementary Material

Supplementary video 1

Supplementary video 2

Supplementary video 3

Supplementary Information

## Figures and Tables

**Table 1:** Experimental parameters and calculated dose. The distance *z*_1_ indicates the first distance to which all images are resized in case of multiple distances. As Fig. 2 shows individual projections taken out of the full tomographic scan, the total exposure times must be divided by the number of projections.

	Fig. 2(c)/Fig. 3	Fig. 2(d,f)/Fig. 4	Fig. 2(e,g)/Fig. 5	Fig. 6(a,b)	Fig. 6(c,d)
illumination / beamline	KB / ID22	WG / P10	WG / P10	WG / P10	WG / P10
number of distances	1	1	4	3	5
number of projections	1500	720	900	-	-
total exposure time	150 s	2160 s	3600 s	30 s	5 s
distance 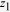 [mm]	299	190	40	39	39
voxel size [nm]	430	245	52	51	51
total fluence [photons/μm^2^]	1.9 · 10^8^	6.9 · 10^6^	2.1 · 10^8^	1.5 · 10^6^	2.5 · 10^5^
total dose [Gy]	5.6 · 10^4^	3.0 · 10^3^	9.1 · 10^4^	633	106
