# Supplementary material for: Phase-contrast zoom tomography reveals precise locations of macrophages in mouse lungs
Source: Sci Rep. 2015 May 12;5:9973. doi: 10.1038/srep09973 (PMC4428069; doi:10.1038/srep09973)
Supplement: Supplementary Information [file srep09973-s4.pdf]

Phase-contrast zoom tomography reveals precise locations of macrophages in mouse lungs: Supplementary Information

Martin Krenkel,[1,*] Andrea Markus,[2] Matthias Bartels,[1]
Christian Dullin,[3] Frauke Alves,[2,3,4] and Tim Salditt[1,†]

[1] *Institute for X-ray physics, University of Göttingen, 37077 Göttingen, Germany*
[2] *Department of Hämatology and Medical Oncology, University Medical Center Göttingen, 37075 Göttingen, Germany*
[3] *Department of Diagnostic and Interventional Radiology, University Medical Center Göttingen, 37075 Göttingen, Germany*
[4] *Department of Molecular Biology of Neuronal Signals, Max-Planck-Institute of Experimental Medicine, 37077 Göttingen, Germany*

SUPPLEMENTARY METHODS 1: PHASE-RETRIEVAL

Propagation-based phase-contrast

Propagation based phase-contrast imaging is based on the conversion of the phase shift imposed by an object on a (partially) coherent illumination wavefront by free space propagation. Depending on the geometric parameters and the resolution, measurable intensity modulations resulting from self-interference of the exit wave can then be recorded. If the projection approximation holds, i.e. if the sample is thin enough not to undergo internal scattering, the phase distribution of the wave directly behind the object (exit wave) is proportional to the projected electron density. An expression for the propagated wave can easily be derived from the angular spectrum method [1] yielding the following convolution type propagation formula

$$\psi(\mathbf{r}_\perp, z) = e^{\mathrm{i}kz}\mathcal{F}_\perp^{-1}\left[\mathrm{e}^{-\mathrm{i}z/2k\left(\xi_x^2+\xi_y^2\right)}\mathcal{F}_\perp\left(\psi(\mathbf{r}_\perp, 0)\right)\right] , \tag{1}$$

where $\psi(\mathbf{r}_\perp, z)$ describes the complex valued scalar wave in a plane perpendicular to the optical axis at a distance z along the optical axis and $\psi(\mathbf{r}_\perp, 0)$ is the initial wave field, e.g. in the exit plane of the object. $\mathcal{F}_\perp$ denotes the Fourier transform in the lateral coordinates ξ_x and ξ_y. Using this equation, it can be easily shown, that the illumination by a spherical wave (leading to a divergent beam) is up to a geometric transform equivalent to the illumination with a plane wave [2]. The effective propagation distance $z_{\text{eff}} = z_{12}/M$ and the effective pixel sizes of the detector $p_{\text{eff}} = p/M$ can be calculated as a function of the geometric magnification $M = (z_1 + z_2)/z_1$ where p denotes the physical pixel size of the detector, z_1 the source-sample distance and z_2 the sample detector distance (see Fig. 1 (a) in the main text).

Phase-retrieval based on the TIE

The most commonly used phase-retrieval techniques are based on the Transport of Intensity Equation (TIE) which is well suited for phase-contrast images recorded at short propagation distance or correspondingly large Fresnel numbers. If only a single (defocus) image is recorded, additional assumptions on the objects are necessary for reconstruction. The assumption of a fixed proportionality between phase shift and attenuation coefficient, also known as phase attenuation duality [3] leads to the following reconstruction formula

$$\phi = \frac{\kappa}{2} \cdot \ln \mathcal{F}_\perp^{-1}\left(\frac{\mathcal{F}_\perp\left(I/I_0\right)}{\left(1 + \frac{\kappa\Delta z}{2k}|\xi|^2\right)}\right) . \tag{2}$$

* mkrenke@gwdg.de
† tsaldit@gwdg.de

	ID 19 setting	P10, large FOV	P10, zoom	single cells
TIE α	$1.3 \cdot 10^{-3}$	$6 \cdot 10^{-8}$	$7 \cdot 10^{-9}$	-
CTF α_0	-	$6 \cdot 10^{-5}$	$5 \cdot 10^{-4}$	-
CTF α_1	0.01	0.1	0.05	10^{-5}
CTF δ/β	200	-	-	10

Supplementary Table I. Regularization parameters used for the phase-retrieval. For the CTF-based reconstruction, either the assumption of a pure phase object is used together with a regularization α_0 for the low frequencies, or the assumption of a phase attenuation duality is made for which a suitable δ/β ratio is chosen based on visual inspection.

Here $|\xi| = \sqrt{\xi_x^2 + \xi_y^2}$ is the modulus of the coordinate vector in reciprocal space, $\kappa = \delta/\beta$ is the ratio of decrement and imaginary part of the refractive index, Δz the propagation distance, $k = 2\pi/\lambda$ the wavenumber and I the measured intensity divided by the (uniformly) illuminating intensity I_0. Similar to the above equation, but easier to derive is the reconstruction formula for a pure phase object in combination with a regularization for the low spatial frequencies [4]

$$\phi = \frac{k}{z}\mathcal{F}_\perp^{-1}\left(\frac{\mathcal{F}_\perp\left(I/I_0 - 1\right)}{|\xi|^2 + \alpha}\right) . \tag{3}$$

The regularization parameter α can physically be justified by the (neglected) residual absorption which manifests itself at low frequencies. This absorption contradicts the assumption of a pure phase object creating a necessity and providing a physical basis for the regularization parameter α. Since α can thus be identified with the additive term in the denominator of Eq. (2), the structure in Fourier space of the two reconstruction formula is in fact the same. For the comparison between TIE and CTF based reconstructions following below, we used a Tikhonov type regularization for the pure phase-TIE

$$\phi = \frac{k}{z}\mathcal{F}_\perp^{-1}\left(\frac{\mathcal{F}_\perp\left(I/I_0 - 1\right) \cdot |\xi|^2}{|\xi|^4 + \alpha}\right) . \tag{4}$$

For Eq. (3) and Eq. (4) the regularization parameter was chosen as follows: If the parameter α is too small, reconstruction results in blurred images as the low frequencies are amplified too much. Choosing α too large, the reconstruction qualitatively reproduces the measured phase-contrast image as it becomes a simple global scaling for all spatial frequencies. Based on these two limiting cases the regularization parameter was chosen based on visual inspection. The resulting parameters are shown in Tab. I.

CTF based phase-retrieval

Based on the propagation formula in Eq. (1) and the assumption of a slowly varying phase and weak absorption, the so called contrast transfer function (CTF) can be derived [5]

$$\tilde{I}(k_x, k_y, z) = \delta_D + 2\tilde{\phi}\sin\chi - \tilde{\mu}\cos\chi . \tag{5}$$

This equation is an expression for the Fourier transform of the intensity I, where $\delta_D(k_x, k_y)$ is the Dirac delta distribution, $\chi = z/(2k)\left(\xi_x^2 + \xi_y^2\right)$, ϕ and μ the projected phase and amplitude distribution and the ~ denotes the Fourier transform. By introducing an error metric for n images $S = \sum_n \tilde{I}_{\text{exp},n} - \tilde{I}(k_x, k_y, z)$, using Eq. (5) for $\tilde{I}$ and minimizing S with respect to $\tilde{\phi}$ one can derive a reconstruction formula, e.g. for a pure phase object ($\mu = 0$)

$$\tilde{\phi} = \frac{\sum_n \tilde{I}_{\text{exp},n}\sin\chi_n}{\sum_n \sin^2\chi_n + \alpha} . \tag{6}$$

Here, a frequency dependent regularization parameter was added to the denominator to compensate zeros of the $\sin^2\chi_n$ expression. The regularization parameter was chosen such, that for high and low frequencies

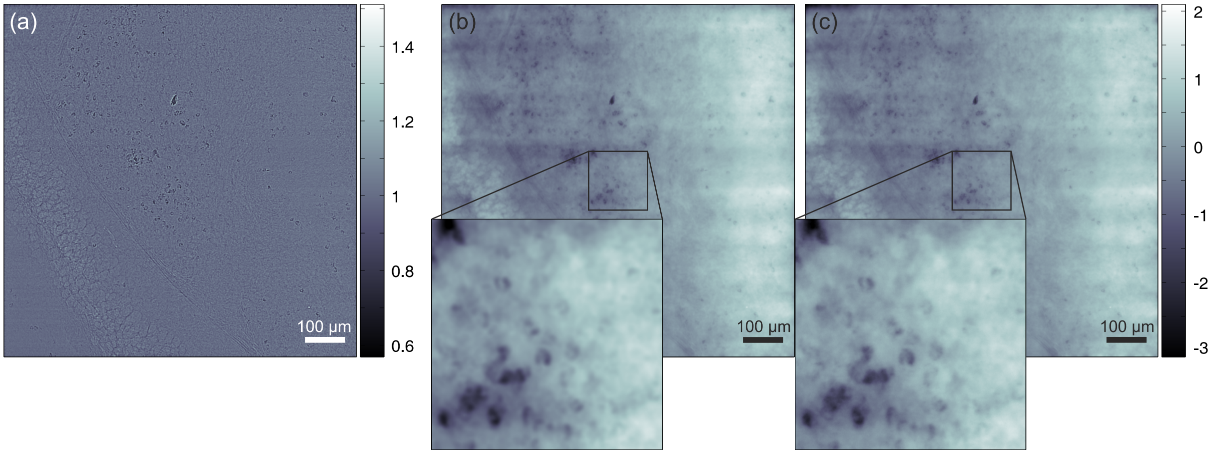

Supplementary Figure 1. Phase-retrieval in the direct contrast regime (ID22 experiment): (a) empty beam-corrected intensity distribution (b) "conventional" TIE phase-retrieval (c) CTF phase-retrieval showing slightly improved resolution. Scalebars denote 100 µm

a value of α_1 and α_0 is applied, respectively, with an error function transition from α_0 to α_1 at the first maximum of the CTF. α_0 corrects the singularities at low frequencies that occur mainly due to residual absorption and beam inhomogeneities, while α_1 corrects the zero crossings of the CTF. The parameters were chosen based on optical inspection of the resulting image and the resulting values can be seen in Tab. I. The use of multiple distances can improve the reconstruction, as for different Fresnel numbers zero crossings occur at different spatial frequencies. An easy way to achieve different Fresnel numbers is to move the sample in the defocused beam, which leads to slightly different magnifications in a cone beam geometry. Here, we rescaled the images based on the experimentally determined distances z_{01} and z_{02} by using an bicubic interpolation with antialiasing implemented in Matlab. To register images in different planes, each image was reconstructed assuming that only one distance was measured and these rough estimates were used to determine the shifts of the individual images to each other by using an algorithm for sub-pixel registration based on correlations [6]. The shifts were used to correct the rescaled intensity distributions with the different defocus distance by applying a phase factor in fourier space. Subsequently, the final phase was retrieved by utilizing Eq. (6).

The use of the CTF based phase-retrieval improves the resolution compared to the TIE phase-retrieval, especially for small Fresnel numbers as can be seen in Fig. 1 and Fig. 2. As CTF-reconstructions yield better results and contrary to current practice, are also well suited for data recorded at a single defocus distance (single image acquisitions), just as the TIE approach, we found no reason to use TIE based reconstruction, unless the assumption of a weak object is violated.

Iterative phase-retrieval

The derivation of the CTF assumes a weakly absorbing object and a slowly varying phase. Especially in the case of a single cell fed with barium particles, the assumptions imposed in the CTF based phase-retrieval are invalid and result in halo-like artifacts around the dense particles which can be seen in the reconstruction in Fig. 3 (c) and (d) which is based on measuring the intensity distribution in eight different planes. An alternative reconstruction method is based on iterative propagation between object and detection planes, with sequential projection onto the different constraint sets (modulus constraint corresponding to the square root of the measured intensities, object constraints). Since the empty beam-corrected intensities still showed some residual low frequency variations which do not stem from the object, these artifacts had to be suppressed as they cause inconsistencies between the individual measurement planes. A suitable correction was implemented by calculating image profiles in areas devoid of Barium labeled cells and dividing the image by a combination of these profiles along with a Fourier filter which suppresses the very low frequencies. An exemplified empty beam-corrected image is shown in Fig. 3 (a) before correction and in Fig. 3 (b) after

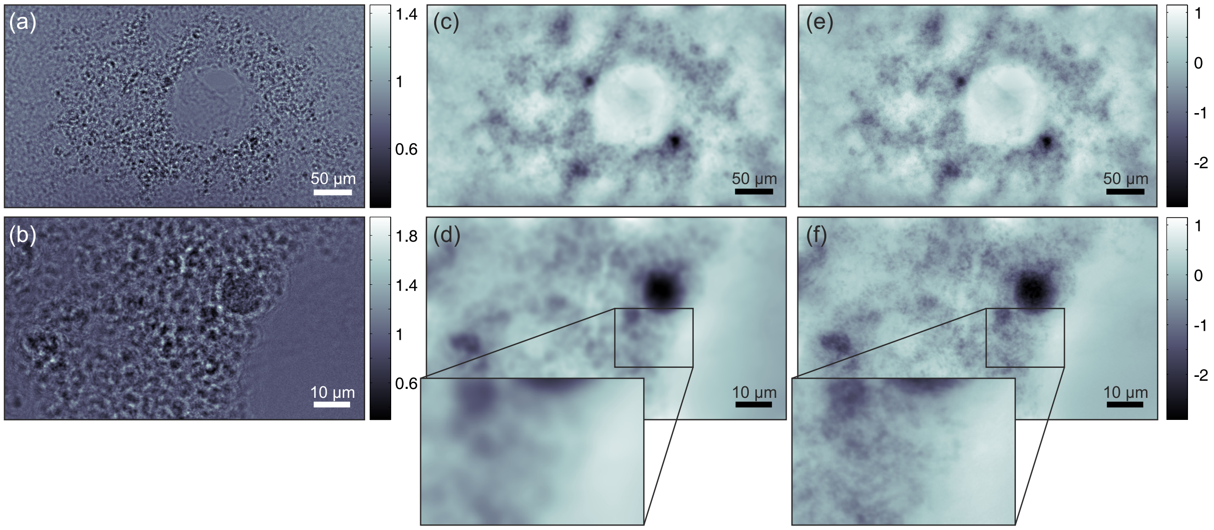

Supplementary Figure 2. Phase-retrieval in the more holographic regime (P10 experiments): (a,b) empty beam-corrected intensity distribution for large field of view (FOV) and zoomed dataset, respectively. (c,d) "conventional" TIE phase-retrieval (e,f) CTF phase-retrieval showing significantly finer structural details, as the TIE can only reconstruct the low frequency components well. In (f) four distances were used to compensate the missing contrast of zero crossings for high spatial frequencies. All other reconstructions are based on a single image. Scalebars denote 50 µm in the upper row (a,c,e) and 10 µm in the lower row (b,d,f).

correction together with a single pixel line profile through the indicated line.

Starting with a random initial guess, the wave function ψ was propagated to a measurement plane and the wavefunction was updated such that it satisfied the measured intensities and kept the current guess for the phases. The updated wave function was subsequently propagated back to the object plane, where positivity of the refractive index decrement δ and β are enforced by setting $|\psi| \leq 1$ and $arg\,(\psi) < 0$ as in [7]. This step was repeated for each measurement plane, all of which have slightly different Fresnel numbers, and the total outer loop was repeated 5000 times. The scheme can be considered a multi-plane Gerchberg-Saxton algorithm, but with the modification that the modulus constraint in the object plane is relaxed to a positivity constraint, and is thus applicable to a much wider range of samples. The resulting phase is shown in Fig. 3 (e) and (f) for different contrast settings.

As the results show, the halo-like artifacts introduced by the CTF reconstruction are not present in the iterative reconstruction. However, the need of a very homogeneous background makes it difficult to apply the iterative reconstruction for a complete tomography data set (in which empty beam images are not available for all projection angles). This difficulty was particularly prominent for region-of-interest (ROI) tomography.

SUPPLEMENTARY METHODS 2: IMAGE PROCESSING

The empty beam-correction

Each recorded image was divided by an empty beam image to reduce the influence of the illumination profile. It is known, that this empty beam-correction is mathematically incorrect and thus small artifacts may be introduced in the intensity distributions [8] which are amplified by phase-retrieval techniques. In the experiments this can be especially seen in the case of a pure KB beam illumination, which has many high frequency artifacts (compare Fig. (1) (c) to Fig. (2) (e) and (f)). Approaches to circumvent the flawed empty beam-division by simultaneously reconstructing the illuminating beam from the measurement along with the object have been proposed [9, 10], but require additional data recorded by scanning. Here we used the fact, that x-ray waveguides provide a very clean illuminating beam, consisting only of a few optical modes. Thus, the illuminating beam is much more uniform and higher resolutions can be achieved although empty

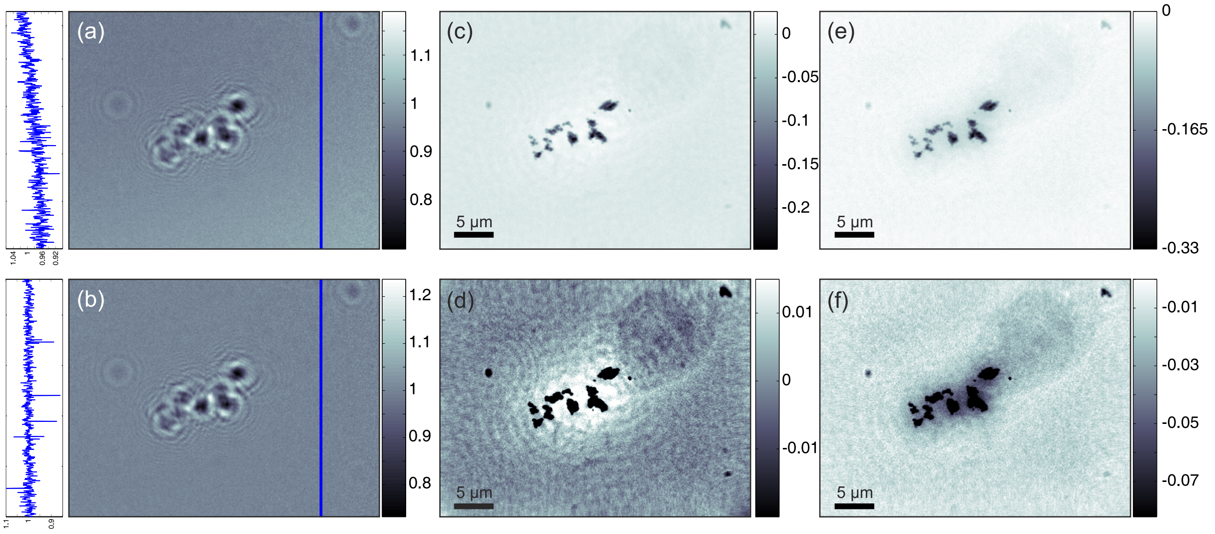

Supplementary Figure 3. Phase-retrieval for single cells in the holographic regime: (a) Example of an empty beam-corrected intensity distribution. Spurious low frequency variations are still present as illustrated by the intensity profile along the blue line. (b) Intensity after further correction steps (see main text) together with a profile along the blue line. (c) Retrieved phase using the CTF method with seven corrected distances. (d) Same reconstruction as in (c) but with adjusted contrast. (e) Retrieved phase using a multi-plane Gerchberg-Saxton (GS) approach with the same seven distances. (f) Same as (e) with adjusted contrast. All scalebars denote 10 μm.

beam-correction is performed.

Tomographic reconstruction

A standard filtered backprojection algorithm implemented in Matlab was used for tomographic reconstruction of the P10 data. Cone beam effects typically encountered for divergent beam were negligible due to the small divergence angle. During a tomographic scan, several empty images were recorded to compensate beam instabilities. Projections for the same angle were aligned before and after recording a new empty beam image using the algorithm presented in [6]. As all samples were larger than the FOV the 3D reconstructions are results of local tomography. Thus, no additional alignment of different projections was performed for the P10 data as especially for the high zoom setting only a small region of interest (ROI) was regarded and features outside this ROI would have disturbed the alignment. The projections obtained at the ID22 beamline were processed with the online tools available during the beamtime and reconstructed using PyHST.

SUPPLEMENTARY METHODS 3: FOURIER SHELL CORRELATION

The Fourier shell correlation (FSC) is a measure for the resolution of a 3D dataset. To calculate the FSC the reconstruction is splitted into two datasets by taking every second angle. The correlation is then calculated as follows

$$FSC(q) = \frac{< F_1 \cdot F_2^* >}{\sqrt{< |F_1|^2 > \cdot < |F_2|^2 >}} ,$$

where F_1 and F_2 denote the splitted datasets in Fourier space and $< \cdot >$ denotes the 3D angular average. The resolution can then be estimated by an intersection of the FSC with a threshold curve. Different threshold curves are discussed in detail in [11]. The 3σ threshold indicates whether significant information above the noise level has been recorded and the 1/2 bit threshold curve indicates the level which is sufficient for interpretation of the data.

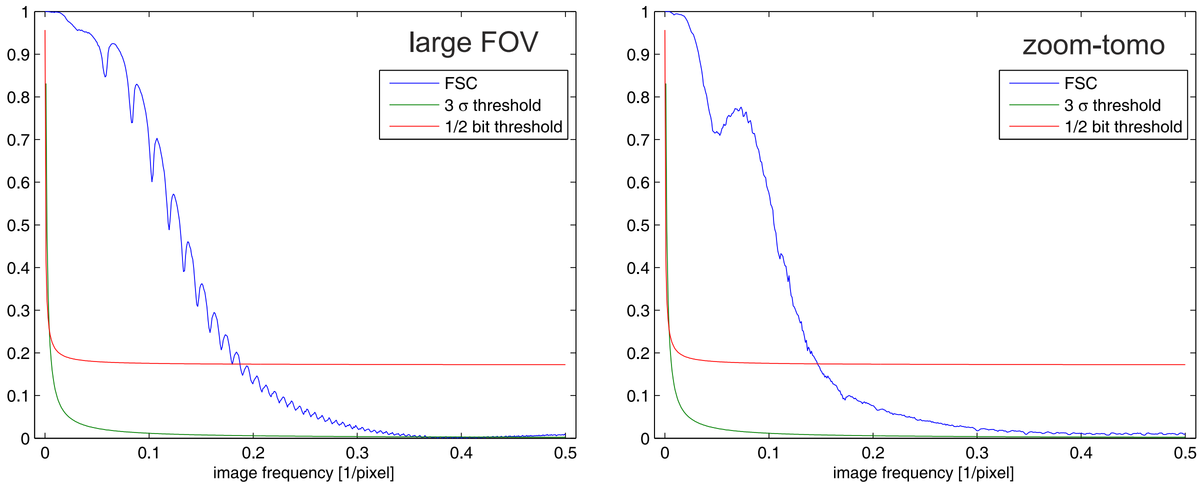

Supplementary Figure 4. Fourier Shell Correlation (FSC) for the large FOV dataset (left) and the zoomed tomography dataset. The intersection with the 1/2 bit threshold corresponds to 648 nm half period resolution for the large FOV dataset and 173 nm for the zoomed dataset.

The resulting FSC curves together with the used threshold curves are shown in Fig. 4. With the effective voxel size of the measurements the resolution of the large FOV dataset could be estimated at 648 nm half period and at 173 nm for the zoomed dataset respectively.

SUPPLEMENTARY DATA 1: RESULTS OF TOMOGRAMS

Figure 5 shows an overview of the different 3D structures obtained from samples measured at the ID 22 beamline. The tomograms were obtained from five different mice where blank denotes a mouse without any instilled macrophages and without asthma. Asthma and control denotes that barium labeled macrophages were given to a mouse with and without asthma, respectively. The renderings show orthoslices through the structure, segmented barium particles which indicate the position of macrophages (green) and selected labeled structures like blood vessels (purple), bronchial tubes (red) and alveolarepithelium (yellow). To label different structures in the reconstructed 3D volumes, automatic threshold based segmentation was used for barium sulfate clusters. Alveolar walls were segmented with a "magic wand" tool followed by application of a watershed algorithm, both available in Avizo (VSG studios). Bronchial tubes and blood vessels were labeled with the contrast based "blow tool" in many slices with subsequent interpolation between the slices.

---

[1] Goodman, J. W. *Introduction to Fourier Optics* (Roberts & Company: Englewood, Colorado, 2005).
[2] Paganin, D. M. *Coherent X-ray Optics* (New York: Oxford University Press, 2006).
[3] Paganin, D., Mayo, S. C., Gureyev, T. E., Miller, P. R. & Wilkins, S. W. Simultaneous phase and amplitude extraction from a single defocused image of a homogeneous object. *J. Microsc.* **206**, 33–40 (2002).
[4] Groso, A., Abela, R. & Stampanoni, M. Implementation of a fast method for high resolution phase contrast tomography. *Opt. Express* **14**, 8103–8110 (2006). URL `http://www.opticsexpress.org/abstract.cfm?URI=oe-14-18-8103`.
[5] Turner, L. *et al.* X-ray phase imaging: Demonstration of extended conditions for homogeneous objects. *Opt. Express* **12**, 2960–2965 (2004). URL `http://www.opticsexpress.org/abstract.cfm?URI=oe-12-13-2960`.
[6] Guizar-Sicairos, M., Thurman, S. T. & Fienup, J. R. Efficient subpixel image registration algorithms. *Opt. Lett.* **33**, 156–158 (2008). URL `http://ol.osa.org/abstract.cfm?URI=ol-33-2-156`.
[7] Ruhlandt, A., Krenkel, M., Bartels, M. & Salditt, T. Three-dimensional phase retrieval in propagation-based phase-contrast imaging. *Phys. Rev. A* **89**, 033847 (2014). URL `http://link.aps.org/doi/10.1103/PhysRevA.89.033847`.

[8] Hagemann, J. *et al.* Reconstruction of wave front and object for inline holography from a set of detection planes. *Opt. Express* **22**, 11552–11569 (2014). URL `http://www.opticsexpress.org/abstract.cfm?URI=oe-22-10-11552`.

[9] Robisch, A.-L. & Salditt, T. Phase retrieval for object and probe using a series of defocus near-field images. *Opt. Express* **21**, 23345–23357 (2013). URL `http://www.opticsexpress.org/abstract.cfm?URI=oe-21-20-23345`.

[10] M. Stockmar, I. Z. B. E. M. D. F. P. P. T., P. Cloetens. Near-field ptychography: phase retrieval for inline holography using a structured illumination. *Sci. Rep.* **3** (2013).

[11] van Heel, M. & Schatz, M. Fourier shell correlation threshold criteria. *J. Struct. Biol.* **151**, 250 – 262 (2005). URL `http://www.sciencedirect.com/science/article/pii/S1047847705001292`.

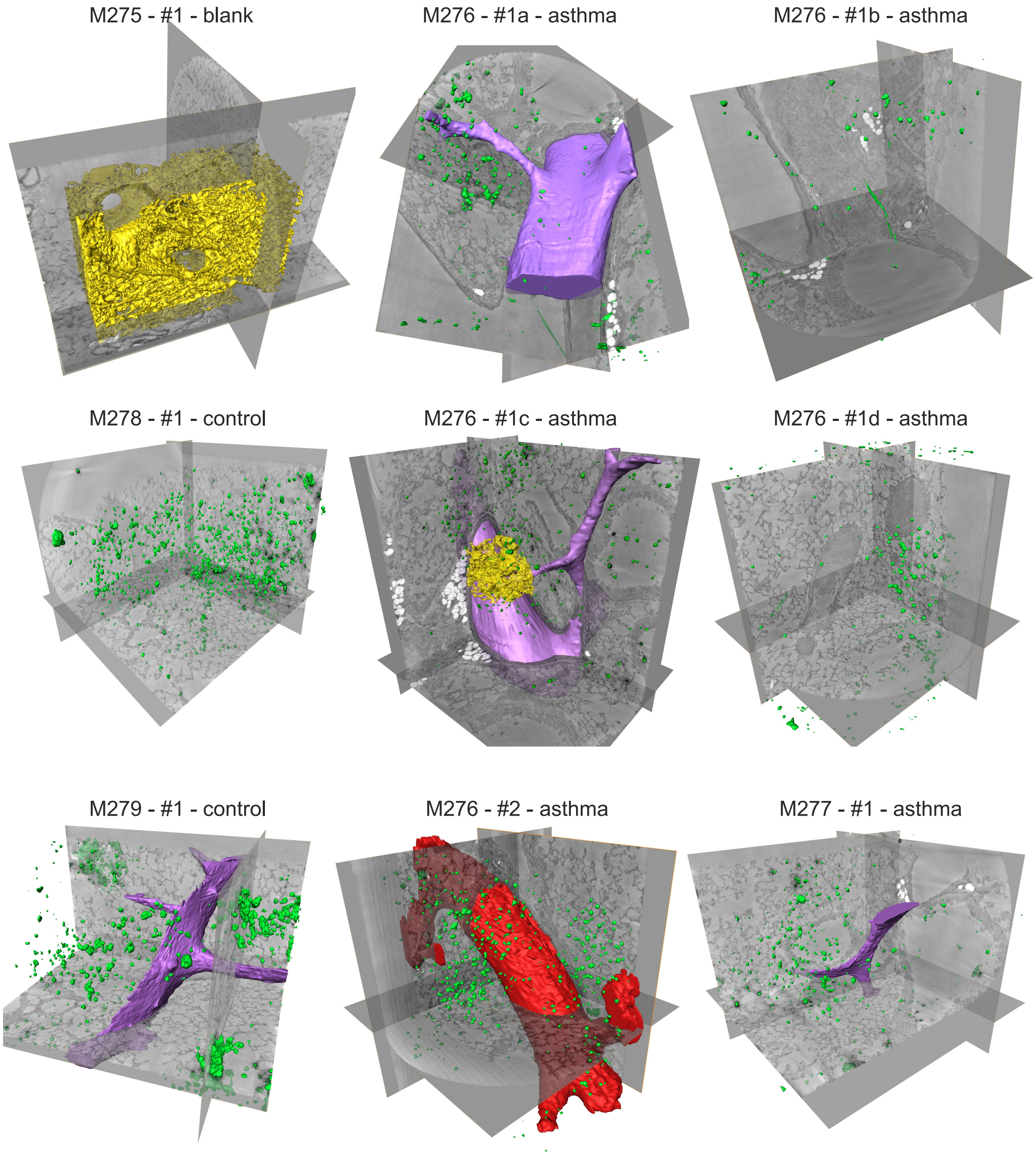

Supplementary Figure 5. Overview of measurements at ESRF. Each view shows a snapshot of three orthoslices and automatically segmented barium particles which indicate the position of macrophages (green). In some volumes, blood vessels (purple), alveolar / bronchial walls (yellow) and bronchial tubes (red) are marked, too. The numbers indicate the mouse (Mxxx) and which slice was used. For the first slice (#1) of mouse M276 four different positions were measured indicated by 1a to 1d. Each FOV covered approximately $800 \times 800 \times 800$ µm^3.
